# Serine Incorporator 2 (SERINC2) Expression Predicts an Unfavorable Prognosis of Low-Grade Glioma (LGG): Evidence from Bioinformatics Analysis

**DOI:** 10.1007/s12031-020-01620-w

**Published:** 2020-07-08

**Authors:** Chunxiao Qi, Lei Lei, Jinqu Hu, Gang Wang, Jiyuan Liu, Shaowu Ou

**Affiliations:** 1 Department of Neurosurgery, The First Hospital of China Medical University, Shenyang, 110001 Liaoning China; 2 Department of Neurosurgery, The Second Hospital of Dalian Medical University, Dalian, 116027 Liaoning China; 3 Department of Rheumatology and Immunology, Dalian Municipal Central Hospital Affiliated of Dalian Medical University, Dalian, 116033 Liaoning China

**Keywords:** Glioma, Serine incorporator 2, Prognosis, Biomarker

## Abstract

Serine Incorporator 2 (SERINC2) is a transmembrane protein that incorporates serine into membrane lipids. The function of SERINC2 in tumors has been reported, but the role of SERINC2 in gliomas is not fully understood. RNA-sequencing data from The Cancer Genome Atlas (TCGA) (530 cases of low-grade glioma (LGG) and 173 cases of glioblastoma multiforme (GBM)) and microarray data from Gene Expression Omnibus (GEO) (Accession No. GSE16011, 284 cases gliomas were included) were acquired. Bioinformatics analysis was performed as the primary method to examine the function of SERINC2 and its correlated genes in glioma. SERINC2 was highly expressed in GBM compared with LGG and normal brain tissues. Elevated SERINC2 expression predicted shorter 5-, 10-, and 15-year overall survival (OS) of LGG patients and isocitrate dehydrogenase-1 (IDH-1) mutation-type LGG patients but had no effect on the OS of GBM patients. Cox regression analysis showed that SERINC2 was an independent factor in LGG OS. Methylation analysis found that 13 CpG methylation sites (methylation450k) correlated with SERINC2 expression in LGG. The mRNA expression level of SERINC2 was significant lower in the DNA deletion group than in the intact and amplification groups. A total of 390 copositive and 244 conegative correlation genes with SERINC2 were obtained from LGG in TCGA-LGG and GSE16011. Gene ontology (GO) category and Kyoto Encyclopedia of Genes and Genomes (KEGG) pathway analyses showed that the copositive correlation genes were primarily enriched in the mitotic process and cell cycle. Combining the results from the protein-protein interaction (PPI) network of SERINC2 correlation genes and CytoHubba led to the selection of 10 hub genes (CDC20, FN1, AURKB, AURKA, KIF2C, BIRC5, CCNB2, UBE2C, CCNA2, and CENPE). OncoLnc analysis confirmed that high expression levels of these hub genes were associated with poor OS in LGG. Our results suggested that aberrant SERINC2 expression existed in glioma and that its expression might be a potential prognostic marker in LGG patients. CDC20, FN1, AURKB, AURKA, KIF2C, BIRC5, CCNB2, UBE2C, CCNA2, and CENPE may be potential biomarkers and therapeutic targets for LGG.

## Introduction

Glioma is the most common primary malignant brain tumor, and it is derived from glial cells with a high infiltrative potency. The WHO classification system divides glioma into four grades, and GBM, the WHO IV grade glioma, has the worst prognosis (Louis et al. 2007). With the development of molecular biological techniques, high-throughput sequencing and microarray techniques have been used to better examine the molecular events of glioma. The 2016 WHO classification generated a combination of the histological and molecular characteristics of glioma, including IDH-1 mutation and 1p/19q codeletion (1p19q co-del) (Louis et al. 2016). Although these findings improved the accuracy of diagnosis and provided new insight into glioma treatment, therapeutic strategies for this infiltrative tumor remain limited (Incekara et al. 2019). Because it is characterized as heterogeneous, the same WHO grade glioma exhibits distinct molecular features and different prognosis (Kloosterhof et al. 2013). The search for specific and effective biomarkers for glioma is necessary.

SERINC2 is a transmembrane protein that belongs to the SERINC family, and it functions to transform serine into lipid membranes during synthesis (Inuzuka et al. 2005). With highly conserved characterization in mammal cells, no amino acid homology of SERINC has been found (Inuzuka et al. 2005). SERINC plays essential roles in regulating the biosynthesis of multiple membrane lipids, such as phosphatidylserine and sphingolipid molecules (Ren et al. 2014). Phosphatidylserine and sphingolipids play critical roles in tumorigenesis and cancer progression (Chang et al. 2000; He et al. 2009; Lee et al. 2012). Phosphatidylserine is induced by scramblase to be exposed on the surface of glioma cells from the normal expression type of an intramembrane model, and this process promotes the phagocytosis of microglial cells (Lee et al. 2012). Irradiation can induce phosphatidylserine exposure on the surface of the vascular endothelium, and the presence of exposed phosphatidylserine enhanced the anticancer effect of an antivascularization drug in an in vivo experiment (Chang et al. 2000). Interference with distinct steps of sphingolipid synthesis and signaling attenuates the proliferation of glioma cells (Bernhart et al. 2015). Knockdown of SERINC2 inhibits the proliferation, migration, and invasion in lung adenocarcinoma (Zeng et al. 2018). Kainite-induced seizures rapidly upregulates SERINC2 in neuronal cell layers of the rat hippocampus (Ren et al. 2014). The roles of SERINC2 in glioma have not been reported. The present study used data mining of the TCGA and GEO datasets to examine the effect of SERINC2 expression on glioma patients’ survival and its potential regulation mechanism. We created a PPI network using correlation genes with SERINC2 to investigate the underlying mechanisms involved in glioma tumorigenesis.

## Materials and Methods

### Data Collection and Bioinformatics Analysis

The present study was based on RNA sequencing data from TCGA-LGG (530 cases, Illumina HiSeq 2000 RNA Sequencing platform) and TCGA-GBM (173 cases, Illumina HiSeq 2000 RNA Sequencing platform) and microarray data from GSE16011, which contains 8 cases of normal brain tissue as a control group, 159 cases of GBM, and 117 cases of low-grade gliomas (GPL8542 platform, Affymetrix GeneChip Human Genome U133 Plus 2.0 Array). Data from TCGA-LGG and TCGA-GBM were obtained via the UCSC Xena browser (https://xenabrowser.net); data from GSE16011 were obtained via the R2 web-based platform: genomics analysis and visualization platform (http://r2.amc.nl). The protein levels of SERINC2 expression in normal brain tissues and gliomas were examined using immunohistochemistry (IHC) and data from the online tool of Human Protein Atlas (HPA) (www.proteinatlas.org) (Uhlen et al. 2015; Uhlen et al. 2010).

The effect of SERINC2 expression on OS of glioma patients was evaluated using Kaplan-Meier curves, which were generated on the R2 platform (GSE16011) and UCSC Xena browser (TCGA-LGG and TCGA-GBM). Data were divided into high and low expression groups based on the median value of SERINC2 expression. Cases without relevant data were eliminated. Cox coefficients were examined via data mining of 21 TCGA cancer types using OncoLnc (http://www.oncolnc.org). Correlation genes (|Pearson *r* | ≥ 0.3) with SERINC2 in LGG were generated via the GlioVis portal (http://gliovis.bioinfo.cnio.es/) (Bowman et al. 2017) and R2 platform. Venn diagrams were constructed using the VENNY 2.1 online tool, which is an interactive tool for comparing lists with Venn diagrams (https://bioinfogp.cnb.csic.es/tools/venny/index.html) to select overlapping genes in groups. The PPI network was generated using the STRING database version 10.5 (http://string-db.org/) (combined score > 0.4 as cut-off criterion), and Cytoscape version 3.7.1 was used to visualize the biomolecular interaction networks of correlation genes. The CytoHubba plugin was used to screen hub genes from the PPI network by considering the top 10 nodes in the network, ranked by degree, to be considered hub genes. The function of hub genes on OS of LGG patients was evaluated using the OncoLnc online tool. GO term and KEGG analyses were investigated using DAVID Bioinformatics Resources 6.8 (https://david.ncifcrf.gov/). GraphPad Prism 7 was used to compare the differences in SERINC2 expression between different groups. Column results are shown in scatterplots.

### Statistical Analysis

Kaplan-Meier survival analysis was performed to compare the OS data in the two cohorts. Column analysis was performed using GraphPad Prism 7 through unpaired *t* tests, and ANOVA. Cox regression analysis was used to evaluate the prognostic value of SERINC2 and its correlation genes in gliomas. *P* < 0.05 was considered statistically significant.

## Results

### SERINC2 Expression Correlates with Glioma Malignancy

**Fig. 1:**
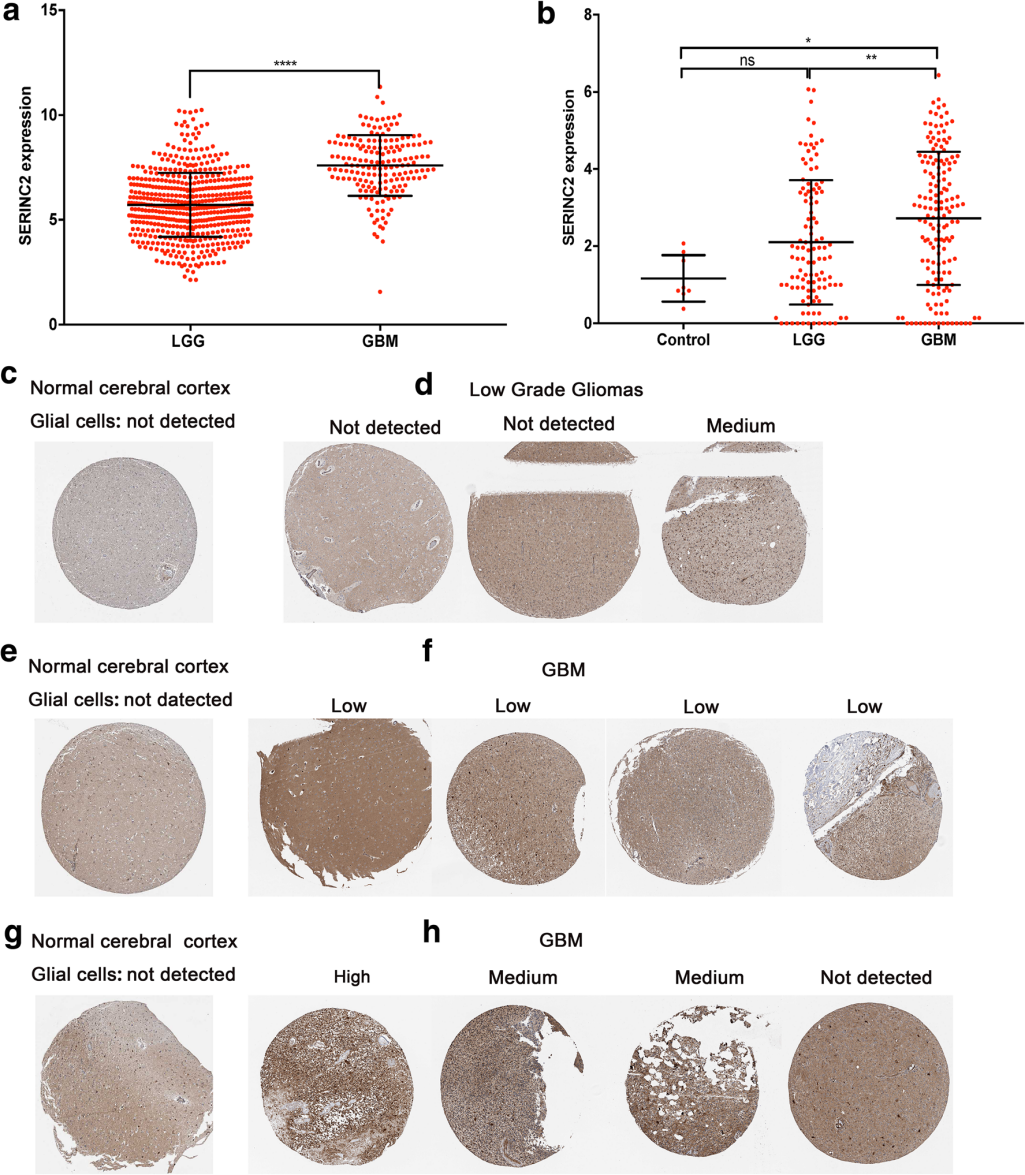
Higher SERINC2 expression correlates with glioma malignancy. **a**, **b** Higher expression of SERINC2 mRNA was found in GBM than in low-grade gliomas and normal brain tissues in the TCGA datasets (**a**) and GSE16011 (**b**). **c, e, g** In normal brain cortex tissues, SERINC2 protein was not detectable in glial cells. **d** One of the 3 LGG cases presented with a medium level of SERINC2 staining, and 2 of the 3 LGG cases were not detectable. **f, h** Seven of the 8 cases of GBM with SERINC2 examined showed positive SERINC2 staining (4 low, 2 medium, and 1 high), and 1 case presented as not detectable. **** *P* < 0.0001; *** *P* < 0.001; ** *P* < 0.01; * *P* < 0.05

Data mining from TCGA showed that SERINC2 expression in 530 cases of LGG was lower than that in 173 cases of GBM. SERINC2 expression in GSE16011 showed that compared with LGG and normal brain tissues, higher expression of SERINC2 occurred in GBM (Fig. 1a–b).

To examine the protein levels of SERINC2 in different grade gliomas and normal brain tissues, we used IHC staining and protein expression scoring in the HPA to examine the level of SERINC2 protein expression in normal brain and glioma tissues (Fig. 1c–h). According to the data from HPA, SERINC2 protein expression was not detectable in glial cells from normal brain tissues (Fig. 1c–g). Seven of the 8 cases of GBM with SERINC2 examined showed positive SERINC2 staining (4 low, 2 medium, and 1 high), and 1 case is presented as not detectable (Fig. 1f, h). One of the 3 LGG cases showed medium staining, and 2 of the 3 cases were not detectable (Fig. 1d). These findings confirmed that SERINC2 was expressed at the protein level in glioma tissues and that GBM had the highest expression.

### High Expression of SERINC2 Predicts Poor Prognosis of LGG

**Fig. 2:**
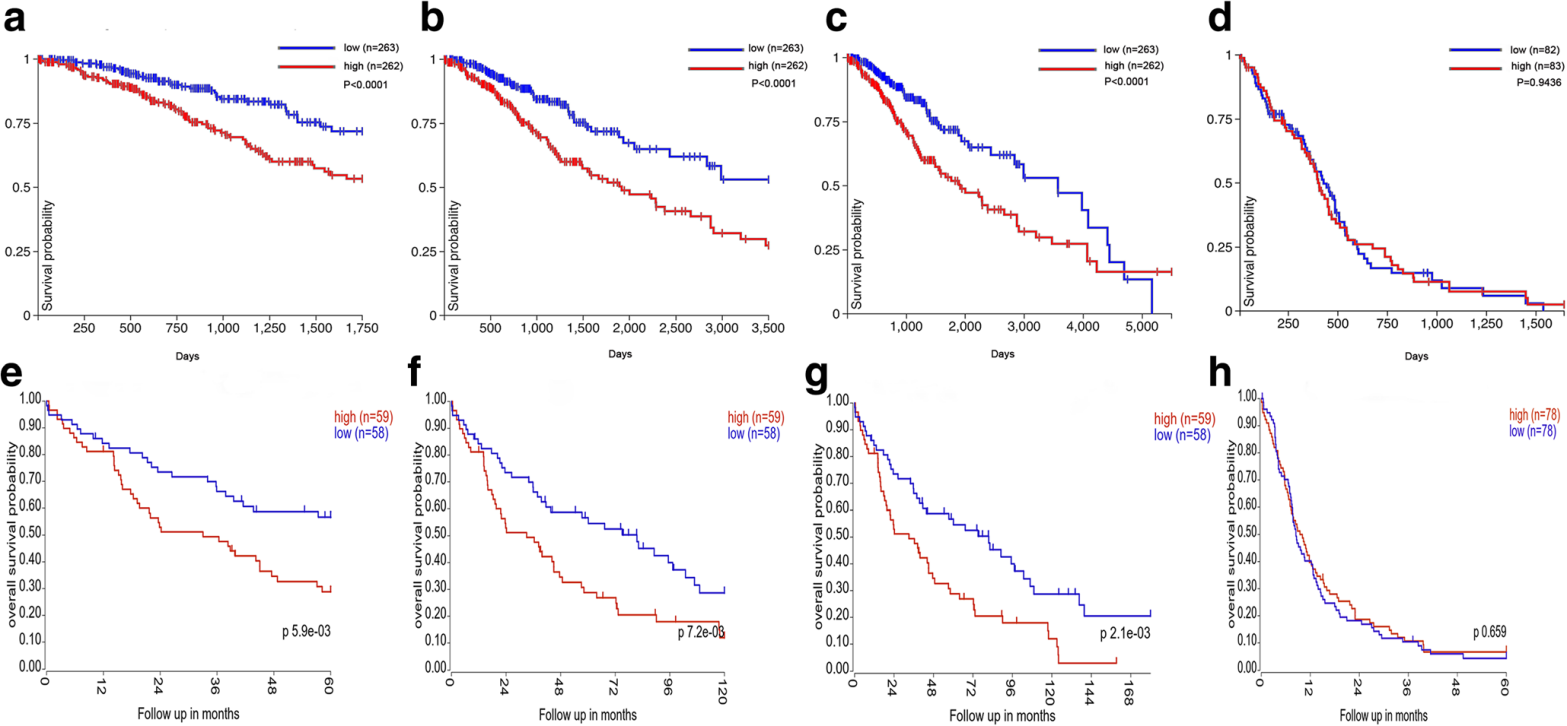
High expression of SERINC2 predicts poor prognosis in LGG but has no effect on OS of GBM patients. **a–c** High expression of SERINC2 showed shorter 5-year (**a**), 10-year (**b**), and 15-year (**c**) OS in the TCGA-LGG dataset, but no significance was found in TCGA-GBM (**d**). **e–h** The same results were found in the GSE16011 dataset; LGG patients with high SERINC2 expression had shorter 5-year (**e**), 10-year (**f**), and 15-year (**g**) OS, and GBM patients showed no significance (**h**)

Data mining of the TCGA datasets using the UCSC Xena browser was used to generate Kaplan-Meier curves in LGG and GBM. We found that higher SERINC2 expression reduced 5-, 10-, and 15-year OS in LGG patients (Fig. 2a–c) but had no effect on OS in GBM patients (Fig. 2d). Consistent results were also found in the GSE16011 dataset, in which higher SERINC2 expression predicted poor 5-, 10-, and 15-year OS in LGG patients (Fig. 2e–g), but no significant results were found in GBM patients (Fig. 2h). The Cox regression results showed that LGG was ranked statistically first among 21 different cancer types based on the FDR correlation, which further confirmed that SERINC2 is an independent predictor of OS in LGG (Table 1).

**Table 1:** Cox regression results of SERINC2 among 21 tumor types

Cancer	Cox coefficient	*P* value	FDR corrected *P* value
LGG	0.412	2.60E-05	1.70E-04
PAAD	0.151	1.60E-01	3.52E-02
SARC	0.224	2.10E-02	1.51E-01
KIRP	− 0.283	5.90E-02	1.65E-01
SKCM	0.138	5.00E-02	1.72E-01
CESC	0.351	9.80E-03	1.75E-01
BLCA	− 0.149	6.40E-02	2.56E-01
LIHC	0.143	1.30E-01	3.65E-01
HNSC	0.120	9.90E-02	4.09E-01
LUAD	0.095	2.40E-01	4.82E-01
COAD	− 0.160	1.20E-01	5.12E-01
KIRC	− 0.071	3.80E-01	5.12E-01
LAML	0.148	1.60E-01	5.54E-01
STAD	0.041	6.30E-01	8.78E-01
LUSC	0.041	5.60E-01	8.86E-01
OV	0.042	6.00E-01	9.21E-01
GBM	− 0.057	5.50E-01	9.28E-01
BRCA	− 0.016	8.50E-01	9.47E-01
UCEC	− 0.212	7.60E-02	9.58E-01
READ	− 0.141	4.70E-01	9.65E-01
ESCA	0.151	3.00E-01	9.77E-01

BLCA, bladder urothelial carcinoma; BRCA, breast invasive carcinoma; CESC, cervical squamous cell carcinoma and endocervical adenocarcinoma; COAD, colon adenocarcinoma; ESCA, esophageal carcinoma; GBM, glioblastoma multiforme; HNSC, head and neck squamous cell carcinoma; KIRC, kidney renal clear cell carcinoma; KIRP, kidney renal papillary cell carcinoma; LAML, acute myeloid leukemia; LGG, brain lower grade glioma; LIHC, liver hepatocellular carcinoma; LUAD, lung adenocarcinoma; LUSC, lung squamous cell carcinoma; OV, ovarian serous cystadenocarcinoma; PAAD, pancreatic adenocarcinoma; READ, rectum adenocarcinoma; SARC, sarcoma; SKCM, skin cutaneous melanoma; STAD, stomach adenocarcinoma; UCEC, uterine corpus endometrial carcinoma

### SERINC2 Expression Is Upregulated in IDH-1 Wild-Type LGG and Predicts Poor Prognosis of IDH-1 Mutation-Type LGG

**Fig. 3:**
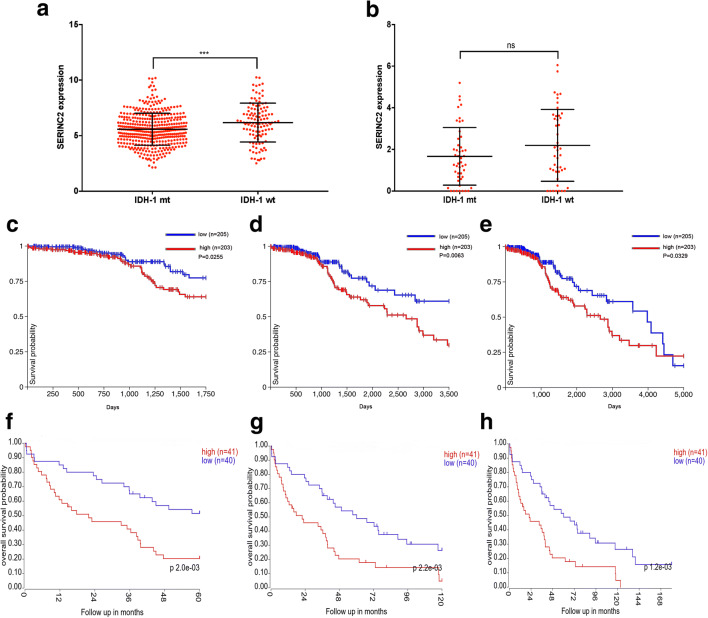
SERINC2 expression correlates with IDH-1 status, and high expression predicts poor prognosis of IDH-1 mutation-type (mt) LGG. **a, b** IDH-1 wild-type (wt) LGG has high expression of SERINC2 in TCGA-LGG (**a**) and GSE16011 (**b**), but no significance was found in the GSE16011 dataset. **c–h** In IDH-1 mt LGG, higher SERINC2 expression showed reduced 5-year (**c**), 10-year (**d**), and 15-year (**e**) OS in TCGA-LGG and GSE16011. **f–h** dataset. **** *P* < 0.0001; *** *P* < 0.001; ** *P* < 0.01; * *P* < 0.05; ns, not significant

We found that SERINC2 was an indicator of OS in LGG. IDH-1 status has been considered an effective factor in predicting OS of glioma patients. We compared SERINC2 expression in different IDH-1 statuses of LGG from GSE16011 and TCGA-LGG. High SERINC2 expression was found in IDH-1 wild-type LGG in TCGA and GSE16011 (Fig. 3a–b), but no statistical significance was found in GSE16011. We examined whether SERINC2 expression affected OS of distinct IDH-1 statuses in LGG. The findings showed that higher SERINC2 expression remarkably reduced the 5-, 10-, and 15-year OS of IDH-1 mutation-type LGG patients in two independent cohorts (Fig. 3c–h). These results indicated that higher SERINC2 predicted IDH-1 wild-type LGG and poor prognosis of IDH-1 mutation-type LGG patients.

### SERINC2 Expression Is Negatively Associated with Its DNA Methylation and Correlates with Alterations of Its DNA Copy Number

**Fig. 4:**
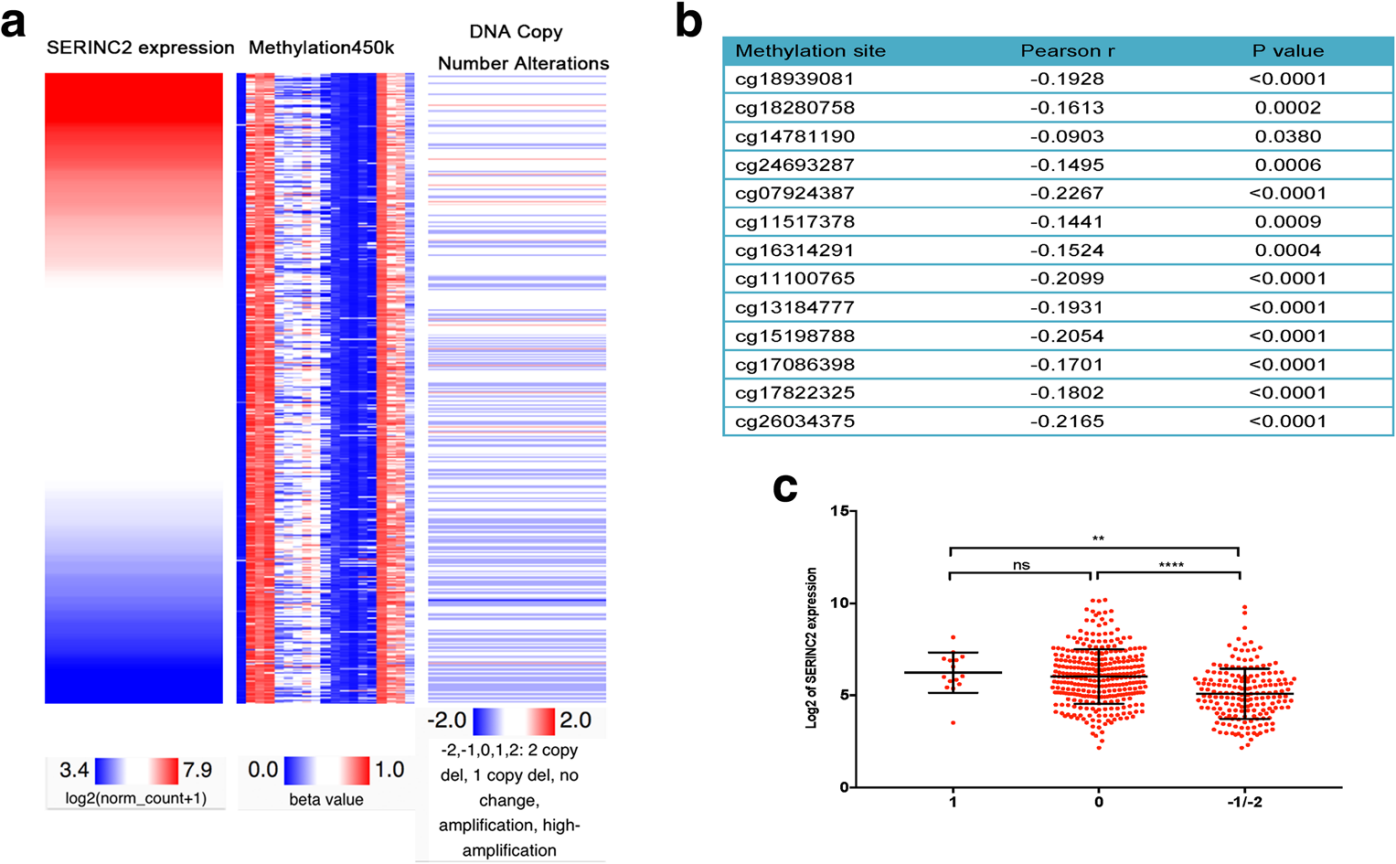
SERINC2 expression correlates with its DNA methylation level and DNA copy number alterations. **a** A comparable heatmap was generated using the UCSC Xena browser to show the correlation between SERINC2 expression and its DNA methylation and copy number alterations. **b** Analysis of methylation 450 k data revealed 13 methylation sites (cg18939081, cg18280758, cg14781190, cg24693287, cg07924387, cg11517378, cg16314291, cg11100765, cg13184777, cg15198788, cg17086398, cg17822325, and cg26034375) that negatively correlated with SERINC2 expression (Pearson *r* < 0, *P* < 0.05). **c** Positive correlation was found between SERINC2 expression and copy number alterations. SERINC2 expression in the DNA copy number amplification group (1) and neutral group (0) was higher than that in the homozygous (− 2) and heterozygous deletion (− 1) groups (*P* < 0.05)

A positive correlation between IDH-1 wild-type LGG and SERINC2 expression was found in TCGA-LGG. Because wild-type IDH-1 is generally associated with hypomethylation status, we checked whether SERINC2 expression correlated with its DNA methylation level. We examined the relationship between SERINC2 expression and its DNA methylation level (Methylation 450 k data) and found 13 methylation sites (cg18939081, cg18280758, cg14781190, cg24693287, cg07924387, cg11517378, cg16314291, cg11100765, cg13184777, cg15198788, cg17086398, cg17822325, and cg26034375) that negatively correlated with SERINC2 expression (*P* < 0.05, Fig. 4a–b). Using genomic data in TCGA-LGG, we further investigated the potential genomic and epigenetic alterations associated with the dysregulation of SERINC2 expression. Comparison of SERINC2 gene expression with DNA copy number alterations of SERINC2 revealed a positive correlation between SERINC2 expression and DNA copy number alterations. SERINC2 expression in the DNA copy number amplification and neutral groups was higher than that in the homozygous and heterozygous deletion groups (*P* < 0.05, Fig. 4c).

### GO and KEGG Pathway Analyses of Correlation Genes with SERINC2 in Two Independent Cohorts

**Fig. 5:**
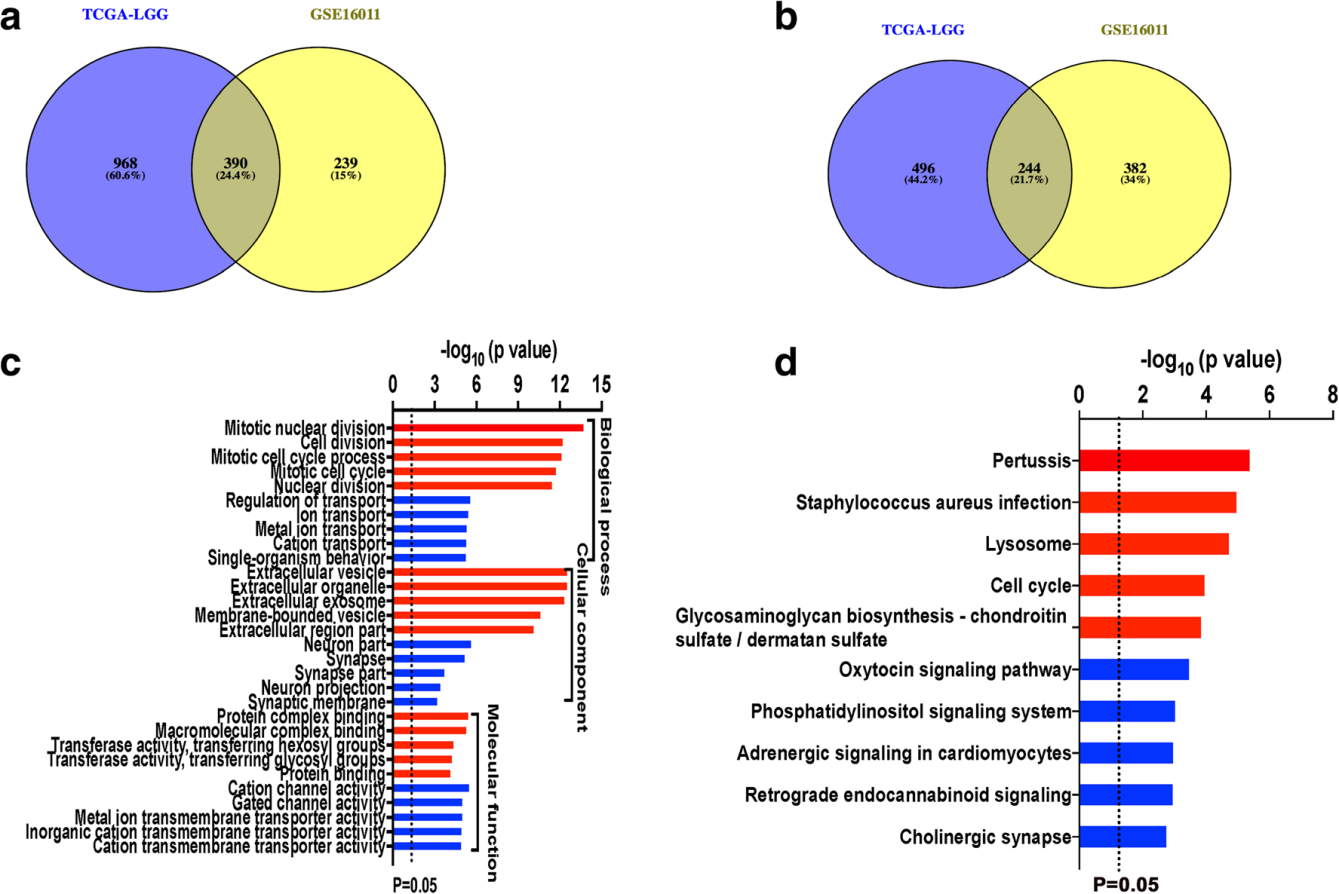
GO and KEGG pathway analyses of correlation genes with SERINC2. **a, b** A total of 1358 and 629 positive correlation genes with SERINC2 were found in TCGA-LGG and GSE16011, respectively, and 390 copositive genes were identified (**a**). A total of 740 and 626 genes were negatively correlated with SERINC2 in the TCGA-LGG and GSE16011 datasets, respectively, and 244 conegative genes were generated (**b**). **c, d** GO and KEGG pathway analyses of the correlation genes. (**c**) Positive correlation genes were enriched in “mitotic nuclear division,” “cell division,” “mitotic cell cycle process,” “mitotic cell cycle,” and “nuclear division.” Negative correlation genes were enriched in “regulation of transport,” “ion transport,” “metal ion transport,” “cation transport,” and “single-organism behavior.” (**d**) The top 5 pathways as ranked by –log_10_(*P* value) are listed. “Red” stands for positive correlation genes, and “blue” stands for negative correlation genes

To further investigate the potential biological function of SERINC2 in LGG, we examined correlation genes with SERINC2 using Pearson coefficient analysis (|Pearson *r*| > = 0.3) in TCGA-LGG and GSE16011. A total of 1358 and 629 positive correlation genes with SERINC2 were found in TCGA-LGG and GSE16011, respectively. Copositive genes were identified as overlapped genes in these two groups, and 390 copositive correlation genes were found (Fig. 5a). For the genes negatively correlated with SERINC2, 740 and 626 genes were found in the TCGA-LGG and GSE16011 datasets, respectively, using the methods described above, and 244 conegative genes were generated (Fig. 5b).

We uploaded the 390 copositive genes and the 244 conegative genes to the online tool DAVID for GO and KEGG pathway analyses separately (Fig. 5c). The top five items, as ranked by their *P* value, are listed. For biological processes (BP), positive correlation genes were enriched in “mitotic nuclear division,” “cell division,” “mitotic cell cycle process,” “mitotic cell cycle,” and “nuclear division.” According to cellular component (CC) analysis, positive correlation genes were primarily enriched in “extracellular vesicle,” “extracellular organelle,” “extracellular exosome,” “membrane-bounded vesicle,” and “extracellular region part.” According to molecular function (MF) analysis, positive correlation genes were enriched in “protein complex binding,” “macromolecular complex binding,” “transferase activity, transferring hexosyl groups,” “transferase activity, transferring glycosyl groups,” and “cytoskeletal protein binding.”

In conegative correlation genes, the top 5 items according to BP analysis were “regulation of transport,” “ion transport,” “metal ion transport,” “cation transport,” and “single-organism behavior.” According to CC analysis, negative correlation genes were enriched in “neuron part,” “synapse,” “synapse part,” “neuron projection,” and “synaptic membrane.” According to MF analysis, negative correlation genes were enriched in “cation channel activity,” “gated channel activity,” “metal ion transmembrane transporter activity,” “inorganic cation transmembrane transporter activity,” and “cation transmembrane transporter activity.”

According to KEGG pathway analysis (Fig. 5d), copositive correlation genes were enriched in “pertussis,” “*Staphylococcus aureus* infection,” “lysosome,” “cell cycle,” and “glycosaminoglycan biosynthesis chondroitin sulfate/dermatan sulfate” and conegative correlation genes were enriched in “oxytocin signaling pathway,” “phosphatidylinositol signaling system”, “adrenergic signaling in cardiomyocytes,” “retrograde endocannabinoid signaling,” and “cholinergic synapse.”

### PPI Network Construction and Hub Gene Selection in Correlation Genes

**Fig. 6:**
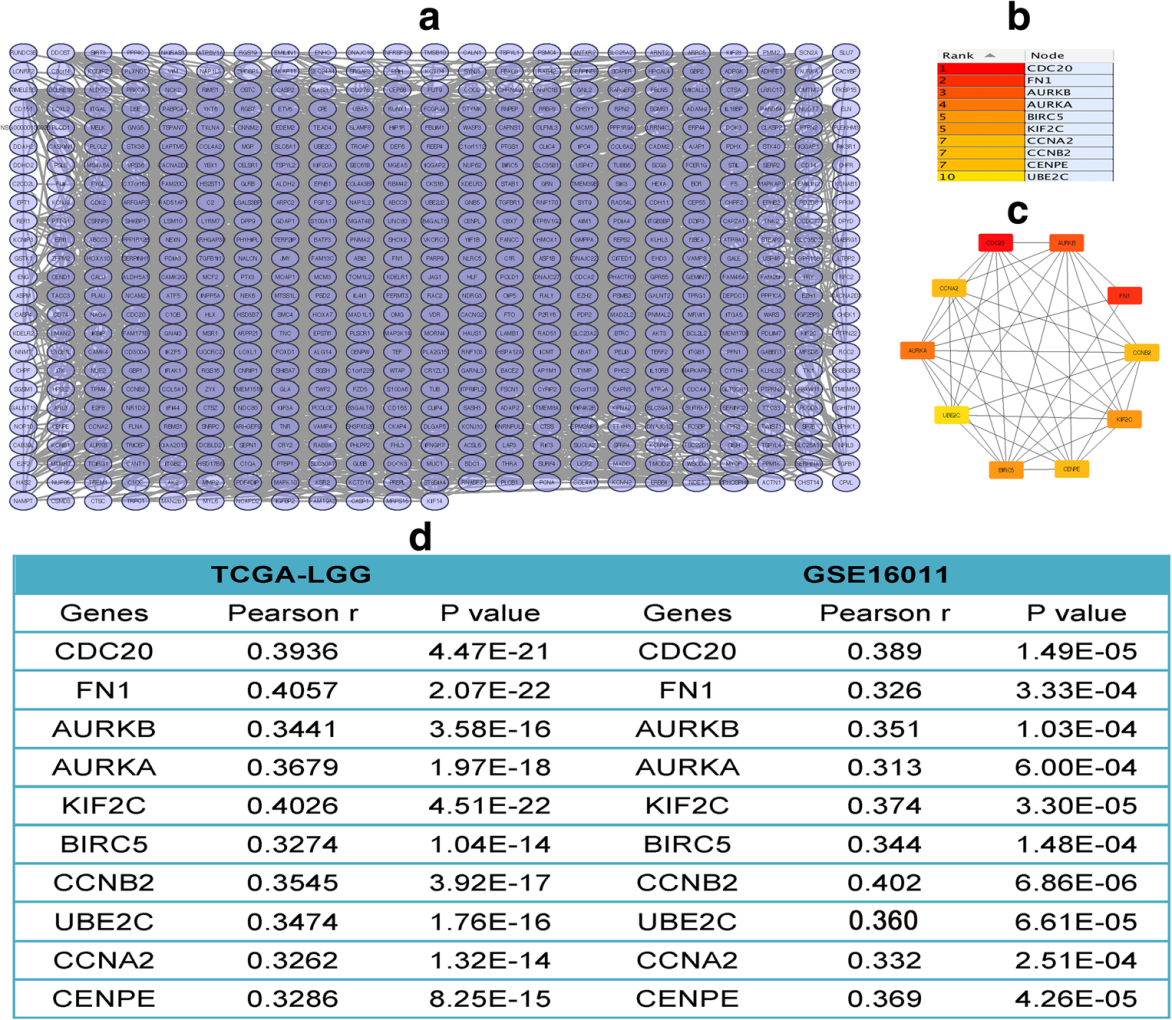
PPI network construction and hub gene selection in correlation genes**. a** A total of 564 nodes and 2801 edges were mapped of the copositive and conegative correlation genes in the PPI network. **b, c** The top 10 genes as ranked by “connectivity degree” in the PPI network were identified as hub genes, including CDC20, FN1, AURKB, AURKA, KIF2C, BIRC5, CCNB2, UBE2C, CCNA2, and CENPE (**b**), and the PPI network of hub genes was created (**c**). All of the hub genes positively correlated with SERINC2 (**d**)

A total of 564 nodes and 2801 edges were mapped for the copositive and conegative correlation genes in the PPI network (Fig. 6a). The Cytoscape and CytoHubba plugins were used to analyze the entire PPI network and select hub genes. The top 10 genes, as ranked by “connectivity degree” in the PPI network, were identified as hub genes, including CDC20, FN1, AURKB, AURKA, KIF2C, BIRC5, CCNB2, UBE2C, CCNA2, and CENPE (Fig. 6b). The PPI network of the hub genes is shown in Fig. 6c. All of the hub genes positively correlated with SERINC2 (|Pearson *r*| > 0.3, *P* < 0.05, Fig. 6d).

### Effect of 10 Hub Genes on OS of LGG

**Fig. 7:**
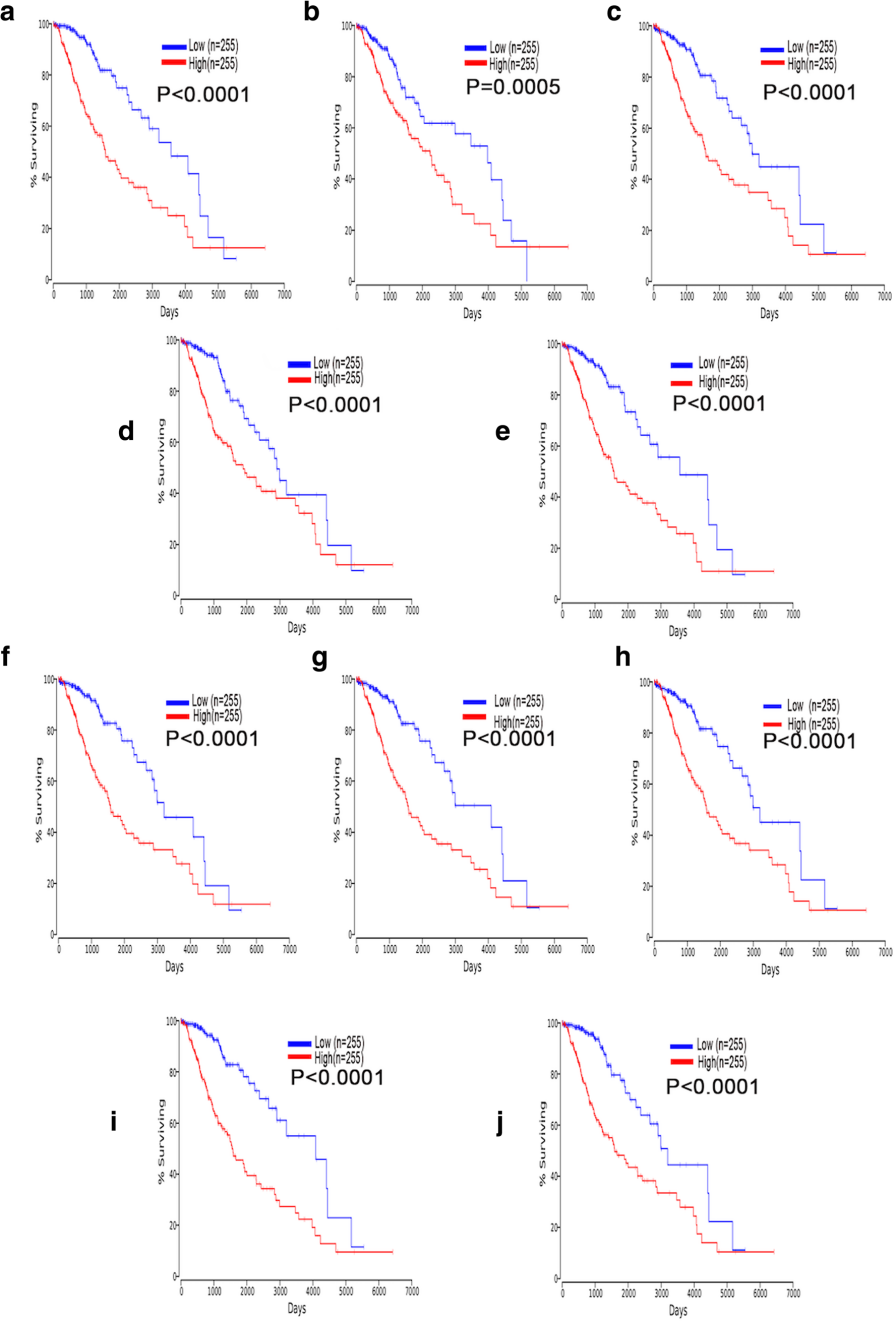
High expression of the hub genes predicts shorter OS of LGG patients. **a–j** Kaplan-Meier curves showed that LGG patients with high expression of CDC20 (**a**), FN1 (**b**), AURKB (**c**), AURKA (**d**), KIF2C (**e**), BIRC5 (**f**), CCNB2 (**g**), UBE2C (**h**), CCNA2 (**i**), and CENPE (**j**) had reduced OS

To examine the function of these hub genes on OS of LGG, we used the OncoLnc online tool to perform Cox regression analysis and generate Kaplan-Meier curves. We found that all 10 hub genes were independent risk factors for evaluating OS of LGG (Table 2), and the Kaplan-Meier curves showed that higher expression of hub genes predicted shorter OS in LGG (Fig. 7a–j).

**Table 2:** Cox regression results of ten hub genes in LGG

Genes	Cox coefficient	*P* value	FDR corrected *P* value
CDC20	0.534	4.80E-07	6.98E-06
FN1	0.309	1.20E-03	4.06E-03
AURKB	0.388	2.00E-04	8.98E-04
AURKA	0.391	3.30E-04	1.36E-03
KIF2C	0.582	6.00E-08	1.35E-06
BIRC5	0.360	3.40E-04	1.39E-03
CCNB2	0.464	8.70E-06	6.87E-05
UBE2C	0.399	1.30E-04	6.31E-04
CCNA2	0.472	4.90E-06	4.37E-05
CENPE	0.542	5.30E-07	7.52E-06

### GO and KEGG Pathway Analyses of Hub Genes

**Fig. 8:**
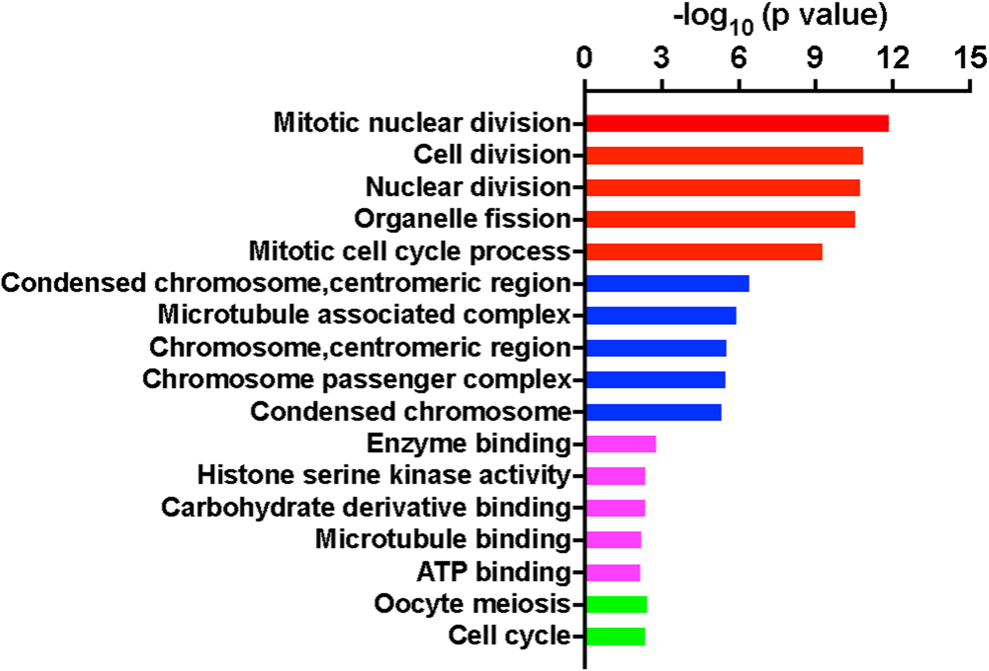
GO and KEGG pathway analyses of the hub genes. “Red” stands for GO-BP, “blue” stands for GO-CC, “magenta” stands for GO-MF. “Green” stands for KEGG pathway analysis. Only two pathways were generated by the hub genes

GO functional enrichment analysis of the hub genes was performed, and the results showed that the hub genes were primarily involved in GO BP terms, such as “mitotic nuclear division,” “cell division,” “nuclear division,” “organelle fission,” and “mitotic cell cycle process.” According to GO CC analysis, these genes were significantly enriched in “condensed chromosome, centromeric region,” “microtubule associated complex,” “chromosome, centromeric region,” “chromosome passenger complex,” and “condensed chromosome.” According to the GO MF terms, these genes were enriched in “enzyme binding,” “histone serine kinase activity,” “carbohydrate derivative binding,” “microtubule binding,” and “ATP binding.” According to KEGG pathway analysis, “oocyte meiosis” and “cell cycle” were the only two pathways generated (Fig. 8).

## Discussion

Glioma is the most common primary malignant brain tumor, and it exhibits highly aggressive features and rapid recurrence after surgery followed by standardized temozolomide (TMZ) chemotherapy and radiotherapy (Incekara et al. 2019). The effects of many molecules on the prognosis of glioma have been confirmed, such as IDH-1 and 1p19q (Louis et al. 2016). However, the heterogeneous nature of glioma makes the function of a single molecule in glioma limited. The present study examined the function of the highly conserved molecule SERINC2 in glioma, and its correlation genes were investigated using bioinformatics analysis.

SERINC2 is a transmembrane protein that functions to transfer serine to lipid membranes during synthesis (Inuzuka et al. 2005). Knockdown of SERINC2 inhibits proliferation, migration, and invasion of lung adenocarcinoma (Zeng et al. 2018). Kainite-induced seizures rapidly upregulate SERINC2 in neuronal cell layers of the rat hippocampus (Ren et al. 2014). Player and colleagues reported the differential distribution of SERINC2 in normal human tissues (Player et al. 2003), and the transcript level of SERINC2 was the highest in the bladder, kidney, and muscle but was undetectable in the brain, spleen, and heart. Our understanding of the role of SERINC2 in cancer is limited and requires further investigation (Zeng et al. 2018).

The present study used the RNA sequencing and microarray data from TCGA and GEO and IHC staining from the HPA and found that the mRNA and protein expression levels of SERINC2 were higher in glioma than in brain tissues and that GBM had the highest SERINC2 expression. Our results are consistent with previous studies that showed that brain tissues had lower expression of SERINC2 (Player et al. 2003). We examined the function of SERINC2 in OS of glioma; although the highest SERINC2 expression was found in GBM, it had no effect on OS of GBM patients. Higher SERINC2 expression predicted shorter 5-, 10-, and 15-year OS in LGG patients, and it was an independent predictor of prognosis in LGG. To examine the underlying mechanism and biological function of this molecule in LGG, we first examined the relationship between SERINC2 and IDH-1 status. IDH-1 status is a reliable biomarker in predicting glioma prognosis, and it correlates with CpG island methylation (Bleeker et al. 2009; Turcan et al. 2012). Higher SERINC2 expression was found in IDH-1 wild-type LGG in the present study. We also found 13 methylation sites in the methylation 450 k data that negatively correlated with SERINC2 expression. Zhou et al. (Zhou et al. 2019) showed that IDH-1 mutation-type glioma had different lipid metabolism compared with IDH-1 wild-type glioma. All the detected differential phosphatidylserine molecules were increased in IDH-1 mutation glioma, but sphingolipid was decreased. SERINC2 plays essential roles in regulating the biosynthesis of multiple membrane lipids, such as phosphatidylserine and sphingolipid (Ren et al. 2014). We hypothesized that IDH-1 status would interfere with the expression and function of SERINC2 in lipid biosynthesis. Although the present study confirmed the importance of SERINC2 in OS of IDH-1 mutation-type LGG patients and the underlying regulation mechanism with DNA methylation, further investigation of SERINC2 expression and its regulation mechanism is needed.

We further investigated the underlying mechanism of SERINC2 in glioma tumorigenesis and generated a list of positive and negative correlation genes with SERINC2 using Pearson correlation analysis. Ren et al. (Ren et al. 2014) reported that the downregulation of SERINC1 expression resulted in impaired cell cycle progression of hepatocellular carcinoma. Zeng et al. (Zeng et al. 2018) found that SERINC2 knockdown inhibited lung adenocarcinoma proliferation, migration, and invasion but without significant differences in the cell cycle. The present study focused on the possible pathways of SERINC2 correlation genes and further analyzed the correlation genes using GO term and KEGG pathway analyses. Positive correlation genes with SERINC2 were significantly enriched in mitotic division and the cell cycle, which are important molecular mechanisms in regulating cancer cell growth and proliferation. However, in vivo and in vitro studies are urgently needed to further elucidate the underlying mechanism of SERINC2 in glioma oncogenesis.

We created a PPI network using SERINC2 correlation genes, and 10 hub genes derived from the PPI network also showed independent function in OS of LGG patients. Therefore, we investigated the potential biological function of these hub genes in LGG, and these hub genes were primarily involved the processes of “mitotic nuclear division,” “cell division,” “nuclear division,” “organelle fission,” and “mitotic cell cycle process.” These results further indicated that SERINC2 is a potential risk biomarker of OS of LGG patients, and further study would be valuable.

The functions of the hub genes derived from the correlation gene network for predicting glioma prognosis were also reported. Overexpression of cell division cycle 20 (CDC20) in glioma enhanced tumor TMZ resistance and reduced OS of glioma patients (Wang et al. 2017; Zhang et al. 2019). Aurora kinase B (AURKB) and ubiquitin-conjugating enzyme E2 C (UBE2C) are involved in the tumorigenesis of gliomas and other malignancies (Alafate et al. 2019), and simultaneously elevated expression of AURKB and UBE2C is strongly correlated with poor prognosis and therapy resistance in glioma. Interactions between aurora kinase A (AURKA) and AURKB stabilize and protect AURKA/B from degradation, and AURKB is closely correlated with chemoresistance and radioresistance (Alafate et al. 2019; Yu et al. 2017). Aberrant kinesin family member 2C (KIF2C) expression in glioma has been confirmed and predicted as a potential independent prognosis marker for glioma patients (Bie et al. 2012). Increased levels of Survivin, which is encoded by the baculoviral IAP repeat containing 5 (BIRC5) gene, are associated with proliferation markers and histological malignancy grade in gliomas and are inversely associated with prognosis and may play vital roles in the adaptive evolution of tumors (Conde et al. 2017). The function of fibronectin 1 (FN1), cyclin A2 (CCNA2), and cyclin B2 (CCNB2) in glioma OS has been reported (Doan et al. 2019; Geng et al. 2018; Long et al. 2017).

The present study used data mining of the TCGA and GEO datasets to characterize the profiles of SERINC2 expression in glioma and investigated the association between the expression level of SERINC2 and OS in glioma. Our results reflected the vital function of SERINC2 in the evaluation of OS in LGG and demonstrated the potential valuable clinical role of SERINC2 in LGG. An interaction network generated by the correlation genes with SERINC2 was used to investigate the potential biological function of these genes, and the hub genes in this network were analyzed in depth. Taken together, our findings identified that SERINC2 and the hub genes in the network may play central roles in the process of LGG.

However, several limitations exist in the present study. Because of technological limitations, we could not further elucidate the underlying mechanisms of SERINC2 in the regulation of OS in LGG and glioma malignancy. Although this study is preliminary, these problems promote future examination of this valuable research.

## Conclusions

In conclusion, our results support the important role of SERINC2 in glioma malignancy and LGG patient prognosis. The hub genes derived from the SERINC2 correlation gene network may be effective biomarkers for evaluating LGG prognosis.
